# Understanding the Reciprocal Interplay Between Antibiotics and Host Immune System: How Can We Improve the Anti-Mycobacterial Activity of Current Drugs to Better Control Tuberculosis?

**DOI:** 10.3389/fimmu.2021.703060

**Published:** 2021-06-28

**Authors:** Hyun-Eui Park, Wonsik Lee, Min-Kyoung Shin, Sung Jae Shin

**Affiliations:** ^1^ Department of Microbiology and Convergence Medical Science, Institute of Health Sciences, College of Medicine, Gyeongsang National University, Jinju, South Korea; ^2^ School of Pharmacy, Sungkyunkwan University, Suwon, South Korea; ^3^ Department of Microbiology, Institute for Immunology and Immunological Diseases, Brain Korea 21 Project for Graduate School of Medical Science, Yonsei University College of Medicine, Seoul, South Korea

**Keywords:** mycobacteria, tuberculosis, anti-TB drug, immune response, Mtb response

## Abstract

Tuberculosis (TB), caused by *Mycobacterium tuberculosis* (Mtb) infection, remains a global health threat despite recent advances and insights into host-pathogen interactions and the identification of diverse pathways that may be novel therapeutic targets for TB treatment. In addition, the emergence and spread of multidrug-resistant Mtb strains led to a low success rate of TB treatments. Thus, novel strategies involving the host immune system that boost the effectiveness of existing antibiotics have been recently suggested to better control TB. However, the lack of comprehensive understanding of the immunomodulatory effects of anti-TB drugs, including first-line drugs and newly introduced antibiotics, on bystander and effector immune cells curtailed the development of effective therapeutic strategies to combat Mtb infection. In this review, we focus on the influence of host immune-mediated stresses, such as lysosomal activation, metabolic changes, oxidative stress, mitochondrial damage, and immune mediators, on the activities of anti-TB drugs. In addition, we discuss how anti-TB drugs facilitate the generation of Mtb populations that are resistant to host immune response or disrupt host immunity. Thus, further understanding the interplay between anti-TB drugs and host immune responses may enhance effective host antimicrobial activities and prevent Mtb tolerance to antibiotic and immune attacks. Finally, this review highlights novel adjunctive therapeutic approaches against Mtb infection for better disease outcomes, shorter treatment duration, and improved treatment efficacy based on reciprocal interactions between current TB antibiotics and host immune cells.

## Introduction

Tuberculosis (TB) is a chronic infectious disease caused by an obligate pathogen, *Mycobacterium tuberculosis* (Mtb), in humans (1). According to the WHO report, in 2020, approximately 10 million people were newly diagnosed, and 1.3 million people died from this notorious disease (2). Moreover, the recent treatment success rate was 82% for drug-sensitive TB and 55% for multidrug-resistant (MDR)-TB (3). There has been a gradual increase in the incidence of MDR-TB, defined as resistance to isoniazid (INH) and rifampicin (RIF), and extensively drug-resistant (XDR)-TB, defined as *in vitro* drug resistance to not only INH and RIF, but also all fluoroquinolones and at least one injectable aminoglycoside (4).

The presence of a mycobacterial population with more than one bacterial phenotype has been observed in patients with TB, as indicated by bacterial populations with varying growth dynamics in sputum samples (3). TB treatment strategy involves long-term treatment with several drugs for at least six months, which may increase the risk of MDR- and XDR-Mtb emergence (4–6), which is attributed to residual bacteria that are sheltered from or unresponsive to antibiotic treatment in heterogenous mycobacterial populations in patients (3). Thus, enhancing treatment success rate, shortening treatment duration, and preventing MDR Mtb emergence are the most critical factors for successful TB treatment. In this review, we provide an understanding of the mechanism underlying the generation of persistent mycobacteria in heterogeneous mycobacteria populations under immune- or drug-induced stress and discuss the effects of anti-TB drugs on host immune responses as opposed to their effects on Mtb. This review provides insights that may contribute to the development of host immune-mediated therapeutic strategies to eliminate persistent mycobacteria more effectively, thereby enhancing treatment success and preventing the development of MDR-TB.

## Mycobacterial Persisters Adapt to Stresses in the Host and Exhibit Antibiotic Tolerance

### Antibiotic Tolerance

Host-related stresses, such as hypoxia, acidic conditions, nutrient starvation, oxidative stress, and cytokine responses, alter the metabolic state of pathogens and eventually induces a drug-tolerant phenotype termed “persister” (7–10). These persister cells can maintain an unreplicated status and simultaneously survive antibiotic treatment. After cessation of anti-TB therapy, the surviving persisters revive their metabolism for replication, subsequently causing a relapse. Thus, antibiotic-tolerant persisters are considered surviving bacteria that did not undergo genetic mutations even after long-term antibiotic treatment (11). Although antibiotic tolerance and antibiotic resistance share common characteristics, they differ in several aspects (12, 13). Antibiotic resistance is generally inheritable and occurs in a drug-specific manner, while antibiotic tolerance is not inheritable and functions broadly. Antibiotic resistance is accompanied by an increase in minimum inhibitory concentration (MIC) of drugs, while antibiotic-tolerant and susceptible subpopulations show identical MIC (13). Tolerance refers only to bactericidal antibiotics and not to bacteriostatic antibiotics, unlike resistance (12).

The mechanism of antibiotic tolerance through the formation of persisters in response to a variety of stresses, including nutrient deprivation, oxidative stress, acidic environment, osmotic conditions, and host immune-mediated stresses, has been described in many pathogenic bacteria, including *Escherichia coli*, *Staphylococcus aureus*, *Pseudomonas aeruginosa*, and Mtb (14–18). Several mechanisms underlying the generation of persisters in response to the stresses have been identified; these include metabolic regulation, such as toxin–antitoxin (TA) systems, stringent and SOS responses, and biofilm formation (19–24). Understanding the mechanisms of persister formation under various stresses and developing therapeutic strategies specifically targeting the mechanisms related to antibiotic tolerance are expected to contribute to TB control. Therefore, here, we review the detailed mechanism of persister formation induced by host-mediated stress in Mtb and its effect on antibiotic tolerance.

### Mtb Adapts to Host-Mediated Stresses Through Metabolic Regulation

#### Regulation of Transcription Factors

Mtb encounters various stresses, such as acidic pH, oxidative stress, hypoxia, nutrient deprivation, and cytokine-mediated effectors, during infection. On detecting such a stressful environment, Mtb reprograms its metabolism, at the transcriptional level, to survive in the niche (25, 26). Bacteria combat environmental stress to induce changes in antibiotic resistance and toxicity through two-component systems (TCSs), consisting of a sensor histidine kinase and a modulator of cytoplasmic response integrated into the inner membrane, as a stress recognition and response system (27, 28). To date, 12 complete TCSs have been identified in Mtb, of which PhoPR, PrrAB, MprAB, NarL, and TcrXY are involved in response to stresses, including pH, macrophage infection, detergents, hypoxia, low iron levels, and starvation (27).

**Figure 1:**
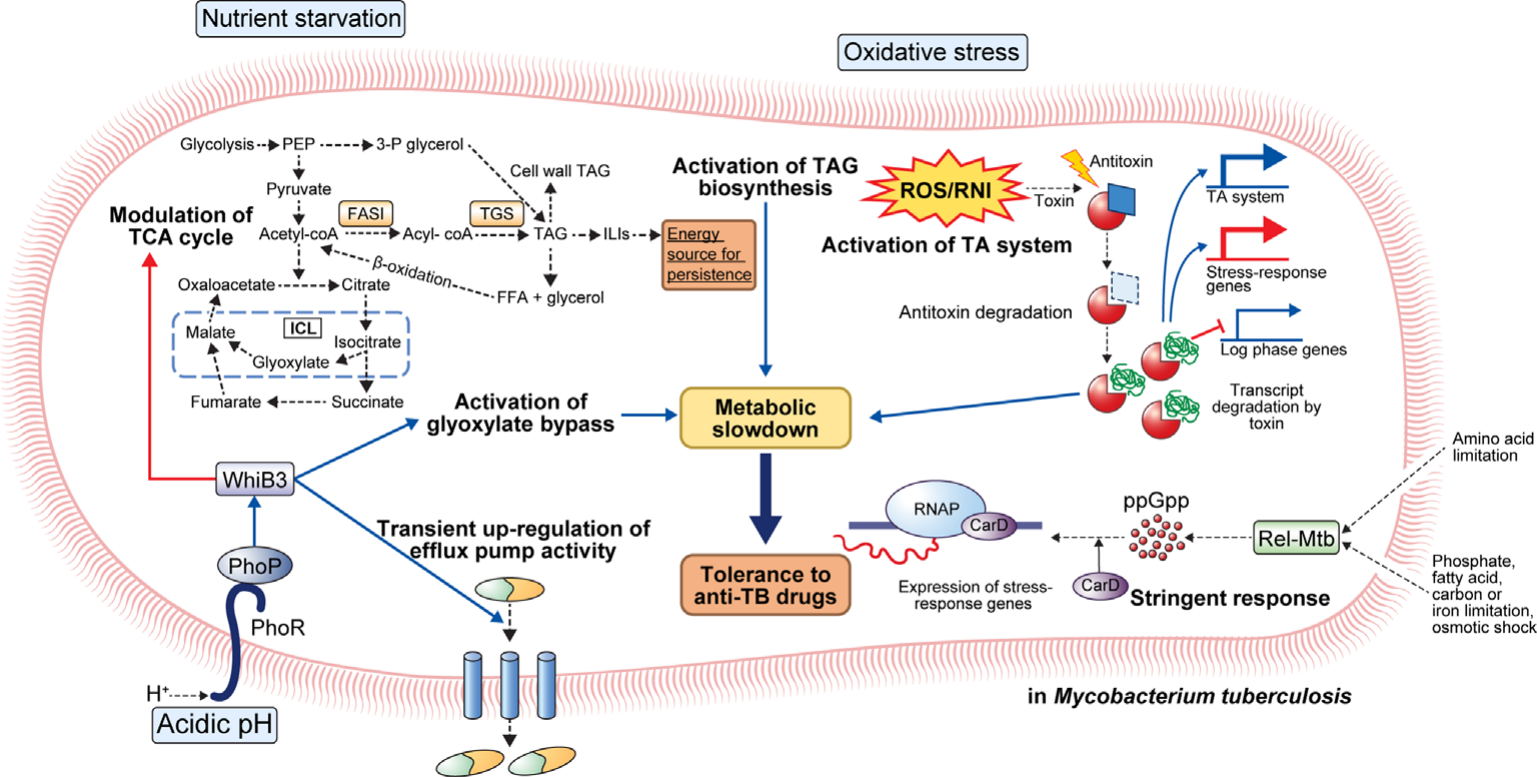
General mechanisms for the establishment of antibiotic tolerance in *Mycobacterium tuberculosis*. Under host-mediated stresses, *M. tuberculosis* (Mtb) adapts to stress conditions via several mechanisms. Under acidic pH, the phoPR two-component system activates transcriptional regulator whiB3 that promotes suppression of the TCA cycle, activation of glyoxylate bypass, and transient upregulation of efflux pump activity. Activation of glyoxylate bypass is mediated by isocitrate lyase that converts isocitrate to glyoxylate under stress conditions. Nutrient starvation induces several changes in Mtb metabolism. Nutrient starvation also suppresses the TCA cycle and activates glyoxylate bypass, thereby enhancing the accumulation of triacylglycerol (TAG). The accumulated TAG is stored in the form of intracellular lipophilic inclusions (ILIs). The stored ILIs are used as an energy source in the persistence state. Additionally, the limitation of amino acids, phosphate, fatty acids, carbon, iron, and osmotic shock induces activation of stringent response through the production of ppGpp by Rel-Mtb. Production of ppGpp activates the expression of stress-response genes that causes a metabolic slowdown. Oxidative stress induces the activation of the TA system. Degradation of antitoxin occurs, and toxin degrades the transcript of log-phase genes. Further, upregulation of stress-response genes occurs, facilitating adaptation to stress conditions. Collectively, the adaptation of Mtb to stress conditions leads to metabolic modulation that results in antibiotic tolerance.

The phoPR TCS may be a critical factor for adaptation to a low pH environment (27). When PhoP detects low pH, it activates the transcriptional regulator whiB3, regulates the expression of pH-responsive gene clusters (*aprABC*, *icl*, *pks2*, *pks3*, *pks4*, and *lipF*), and is involved in the survival of Mtb in macrophages (29–33) (**Figure 1**). Indeed, *phoP* deletion mutants exhibit growth defects in murine bone marrow-derived macrophages (BMDMs) as well as attenuated virulence with reduced bacterial burden in the lungs, liver, and spleen of a mouse Mtb infection model (34). Interestingly, transcriptional analyses revealed an overlapping of the repressed genes in H37Ra and *phoP* knockout mutant of H37Rv (35). Moreover, the incorporation of intact *phoP* into the H37Ra genome increased the bacterial persistence in murine BMDMs (35). In another study, the *phoP* mutant Mtb strain showed considerable attenuation in severe combined immunodeficient mice compared to the parental and BCG strains (32). Moreover, the Mtb *phoP* deletion mutant strain conferred protective anti-TB immunity in mouse and guinea pig models, indicating its potential as a live vaccine candidate (32). Liu et al. demonstrated that the expression of five regulons, DosR, MprA, PhoP, Rv1404, and Rv3058c, is responsible for the antibiotic tolerance of Mtb; these five regulons controlled the expression of over 50% of the upregulated genes after treatment with different anti-TB drugs, and their (DosR, PhoP, and MprA) deletion reduced drug tolerance under stress conditions (36).

#### Stringent Response

The stringent response is a conserved global signaling system that promotes bacterial survival in various environments, such as nutrient deprivation and other stresses (37). Particularly, stringent responses have been reportedly caused by amino acids, carbon, nitrogen, or phosphorus starvation, as well as UV exposure and fatty acid depletion (37). The stringent response is mediated by the hyperphosphorylated guanine nucleotides ppGpp and pppGpp, collectively referred to as [(p)ppGpp], and inorganic polyphosphate [poly(P)], and the synthesized signaling molecules regulate bacterial transcriptional changes under various stress conditions (38, 39). In Mtb, (p)ppGpp synthesis is induced by nutrient deprivation, long-term culture, and chronic infection in animal models, and it has been reported to be necessary for Mtb survival (37, 40, 41). Two proteins, RelA and SpoT, responsible for the synthesis of (p)ppGpp in gram-negative bacteria, have been identified, but many gram-positive bacteria, including mycobacteria, have only one protein (Rel) homologous to both RelA and SpoT (37). Accumulation of (p)ppGpp synthesized by Rel-Mtb and the transcription factor CarD in hostile environments, such as nutrient deficiency and oxidative stress, leads to transcription and translation of stress-responsive genes in Mtb (37) (**Figure 1**).

The protein Rel-Mtb modulates the intracellular (p)ppGpp content by regulating its synthesis and hydrolysis *via* an N-terminal hydrolase and synthetase domain (42). Nutrient starvation induces upregulation of *Rv2583c* (*Rel-Mtb*) that subsequently promotes the production of intracellular (p)ppGpp in Mtb (37). *Rel-Mtb* deletion mutant showed a growth defect in liquid media, and the disrupted growth rate was restored when citrate or phospholipid was used as the sole carbon source *in vitro* (37). A disrupted growth rate can induce antibiotic tolerance to drugs that kill actively growing cells. Recently, Dutta et al. showed that Rel-Mtb deficiency induces disruption of antibiotic tolerance under stress conditions, increasing susceptibility to INH (43). They reported that the nutrient-starved Rel-Mtb mutants showed similar metabolic activity as wild type bacteria growing in nutrient-rich conditions (43). Disruption of Rel-Mtb induced increased susceptibility to INH *in vitro* nutrient starvation and BALB/c mouse models (43). Furthermore, they discovered a Rel-Mtb inhibitor through pharmaceutical library screening that showed a direct cytotoxic effect on antibiotic-tolerant Mtb and synergetic effect with INH activity (43). In Mtb, the polyphosphate kinase PPK1 is responsible for poly(P) synthesis, and the exopolyphosphatases, PPX1 and PPX2, and PPK2 are responsible for poly(P) hydrolysis, thereby regulating cellular poly(P) homeostasis (39). The *ppx1* or *ppk2* deletion mutant strains showed low glycerol-3-phosphate (G3P) and 1-deoxy-xylulose-5-phosphate expression levels in bacterial cells, suggesting downregulated G3P synthesis pathway (39). As a result, the *ppk2* and *ppx1* deletion mutant increased susceptibility to plumbagin and meropenem, and clofazimine, respectively (39). Similarly, the ppk1 deletion mutants showed increased susceptibility to INH, levofloxacin, and RIF (44). These results suggest that (p)ppGpp and poly(P) synthesis and their modulators play important roles in the development of antibiotic resistance *in vivo*.

#### Metabolic Modulation

Numerous acid-inducible genes induce a carbon metabolism shift for microbial persistence in the host macrophages. One such acid-inducible gene encodes isocitrate lyase that converts isocitrate to succinate and glyoxylate (45). Moreover, malate synthase catalyzes malate formation by the addition of acetyl-CoA to glyoxylate (45). Overexpression of isocitrate lyase causes the activation of the glyoxylate shunt, subsequently inducing metabolic shifting; pyruvate, succinate, fumarate, and malate levels were increased while the α-ketoglutarate level was decreased in macrophage infection and low pH culture model (46). The limitation of α-ketoglutarate-derived amino acids and oxaloacetate by glyoxylate shunt activation slows bacterial cell growth and metabolic activity (46). Antibiotics can induce growth and metabolic activity arrest in rapidly growing cells. For example, the antimicrobial effect of INH depends on INH conversion to isonicotinoyl by the catalase-peroxidase katG (3). Converted isonicotinoyl binds NAD^+^ to make isonicotinoyl-NAD that inhibits mycolic acid synthesis, a bacterial cell wall component, subsequently interfering with mycobacterial cell wall integrity (3). Thus, reduced need for cell wall synthesis due to arrested growth and metabolic activity due to acidic stress induces tolerance to INH (3). RIF kills metabolically active cells by binding to RNA polymerase subunit B and interfering with transcription; RIF resistance is usually acquired through mutation in *rpoB* that encodes RNA polymerase B protein (47). However, transient antibiotic tolerance has also been reported in previous studies (48–50). In response to environmental stress, Mtb translates a mutated form of RNA polymerase with a lower affinity to RIF, thereby facilitating the acquisition of transient antibiotic tolerance during antibiotic treatment (50). Collectively, growing evidence suggests that metabolically arrested states induce antibiotic tolerance that prevents the complete sterilization of pathogens. Therefore, to eradicate the antibiotic-tolerant bacterial population, a treatment strategy that reactivates the metabolically arrested bacterial population is needed (**Figure 1**).

#### Modulation of Lipid Metabolism

Several host immune-mediated stresses induce intracellular triacylglycerol (TAG) droplet accumulation in Mtb by TAG synthase activity. For example, TAG synthase upregulation was confirmed in multiple-stress conditions, such as hypoxia, low pH, and low iron (51–54). Accelerated TAG synthesis induces a reduction in TCA flux and subsequently enhances the survival of Mtb in the presence of antibiotics, such as INH, streptomycin, ciprofloxacin, and ethambutol (EMB) (54). Interestingly, antibiotic tolerance due to TAG accumulation can be reversed by modulating carbon fluxes with complete inhibition of TAG synthase *in vitro* and *in vivo* (54). Furthermore, *tgs1* deletion mutants continue to grow under stress conditions while wild type strain stops replicating (54). Kapoor et al. developed an *in vitro* model of human granuloma for pulmonary tuberculosis and discovered unique characteristics of Mtb within the granuloma; Mtb showed dormant phenotypes, including the loss of acid-fastness, accumulation of lipid droplet, transcriptional change of lipid metabolism genes, and tolerance to RIF (51). Moreover, treatment with anti-tumor necrosis factor-alpha (TNF-α) monoclonal antibodies induced resuscitation of Mtb as previously described in human TB (51). Similarly, a multiple-stress model that included low oxygen, high CO_2_, low nutrient, and acidic pH showed arrested growth, acid-fastness loss, TAG, and wax ester accumulation, along with the rise in antibiotic tolerance to INH and RIF in Mtb (52). Interestingly, antibiotic tolerance was diminished in the *tgs1* deletion mutant and restored with the addition of complementation. Furthermore, transcriptome analysis using microarray revealed the achievement of the dormant state showing repression of energy generation, transcription and translation machinery, and induction of stress-responsive genes (52). Recently, Santucci et al. identified the mechanism of TAG accumulation to involve intracytoplasmic lipid inclusions (ILI) induced by carbon excess and nitrogen starvation in *M. smegmatis* and *M. abscessus* (53). They also identified *tgs1*-mediated TAG formation and lipolytic enzyme-mediated TAG breakdown mechanisms. Moreover, they discovered that emergence of antibiotic tolerance against RIF and INH induced by low nitrogen and high ILI environment as previously described (53). Taken together, the importance of TAG synthesis in antibiotic tolerance of Mtb suggests the potential of lipid metabolism-related proteins, such as triacylglycerol synthase and fatty-acyl-CoA reductase, as therapeutic targets for abolishing antibiotic tolerance.

#### Toxin–Antitoxin (TA) System

The TA system comprises a stable toxin that interferes with indispensable cellular metabolism and an unstable antitoxin that blocks the toxin activity during persister formation (55, 56). The TA systems are generally divided into seven classes depending on their mechanism (57). In detail, type I and III antitoxin include an RNA antitoxin that interferes with translocation of the toxin as an antisense RNA (type I) or binding to toxin protein to neutralize the toxin activity (type III). Type II antitoxins are proteins that interfere with the toxins by direct binding to the toxin protein. Type IV antitoxins inhibit toxin activity by attaching to the toxin target, while type V antitoxins degrade the toxin mRNA target directly. Type VI antitoxins bind to the toxin; they do not directly degrade the toxin itself but promote its degradation by ClpXP (56). In the type VII TA system, antitoxin acts as an enzyme for the chemical modification of the toxin and subsequently neutralizes the toxin (57). As a representative example, in the HipBA toxin/antitoxin module, HipA is a toxin that inhibits cell growth and induces persister formation, while HipB is an antitoxin that binds to HipA and acts as a transcription inhibitor of the hipBA operon. In particular, high HipA expression leads to multidrug resistance in *E. coli* (55). Characteristically, Mtb has many TA system-related loci in its genome, and at least 88 TAs have been identified (58). According to Keren et al., 10 TA modules were overexpressed in Mtb persister cells, suggesting that the TA system not only contributes to Mtb virulence but also the formation of bacterial persister cells (8). Further, Torrey et al. revealed that multiple pathways such as lipid biosynthesis, carbon metabolism, TA systems, and transcriptional regulators are involved in Mtb persister formation using transcriptional analysis and whole-genome sequencing of Mtb hip mutant (59). Notably, most of the identified Mtb TA systems were Type II, and these include VapBC, MazEF, YefM/YoeB, RelBE, HigBA, and ParDE (60).

Notably, the VapBC TA family is the most abundant type of TA system encoded by Mtb (60). Several studies have demonstrated that host-mediated stress, such as hypoxia and activated macrophages, induces transcriptional activation of multiple VapBC TA loci (61–63). Hudock et al. identified the transcriptional profile from granuloma samples of active and latent TB patients; the expression of eight dosR regulon members (Rv0080, Rv0081, Rv1736c, Rv1737c, Rv2032, Rv2625c, and Rv2630) along with the induction of four pairs of toxin/antitoxin (vapBC19, vapBC21, vapBC33, and vapBC34) were observed within the granulomas of active and latent TB patients (61). Sharma et al. demonstrated that VapC21 overexpression hinders mycobacterial growth, and co-expression of antitoxin VapB21 reverses this effect (62). Moreover, VapC21 overexpression mutant and Mtb cultured in stress conditions, such as nutrient deprivation and hypoxia, exhibited similar transcriptional profiles (62). Furthermore, VapC21 overexpression resulted in upregulated WhiB7 regulon, inducing antibiotic tolerance to aminoglycosides and EMB (62). Talwar et al. identified the role of VapBC12 TA in persister formation under cholesterol-rich conditions; VapC12 RNase toxin targets *proT* transcript that is indispensable for Mtb growth regulation in a cholesterol-rich environment (63). Therefore, the expression of VapC12 RNase toxin induced the generation of a slow-growing population, and this phenotype occurrence was increased in the presence of cholesterol (63). Interestingly, co-expressing of antitoxin *vapB12* disrupted the vapC12-induced phenotype, while *vapC12* deletion enhanced the immunopathologic severity and lung bacterial burden compared with the wild type strain (63). Recently, Yu et al. demonstrated a phosphorylation-dependent TA system in Mtb (58). Specifically, phosphorylation of TgIT by TakA induces toxicity neutralization and allows bacterial growth (58). In stressful conditions, TgIT activation *via* dephosphorylation promotes bacterial growth inhibition, leading to a non-replicating but viable state (58).

#### SOS Response

Various host-mediated stresses, such as reactive oxygen and nitrogen species, result in DNA damage and subsequently induce a DNA repair mechanism called SOS response (64). The SOS response is controlled by two regulator proteins, RecA and LexA. RecA recognizes damaged single-stranded DNA and induces the proteolysis of LexA repressor leading to the activation of SOS genes (64). Völzing and Brynildsen discovered that DNA repair was essential for the survival of ofloxacin-induced persisters and that delayed DNA repair occurred after ofloxacin treatment (65). Another study indicated that the timing of DNA repair was a key factor for the complete recovery of persisters after ofloxacin treatment. Additionally, nutrient starvation increased the survival rate of *E. coli* to approximately 100%, following ofloxacin treatment (66). These results indicate that changes in post-antibiotic treatment recovery time are critical to the formation of persister and support the notion that interference of DNA damage repair systems could be an effective strategy to eradicate the persister population.

Previous studies showed that stress-response regulons, including SOS response genes, were upregulated in Mtb persisters (8, 67). In mycobacteria, the DNA damage repair system comprises LexA-mediated and ClpR factor-mediated mechanisms (67). Further, DnaE2 polymerase, induced by ROS and NOS produced in the host immune response, contributes to mutations during the DNA repair process (68). Recently, inhibition of DNA gyrase by fluoroquinolone was found to modulate Mtb growth in intracellular and extracellular environments (69). Interestingly, inhibition of DNA gyrase contributes to the drug tolerance *via* RecA/LexA-mediated SOS response (69). Choudhary et al. demonstrated that DNA gyrase knockdown Mtb mutant showed decreased drug susceptibility to RIF 24 and 48 h post-treatment, and a similar pattern was observed following INH and EMB treatment (69). Taken together, these findings indicate that changes in post-antibiotic treatment recovery time are critical to the formation of persisters and support the notion that interference by DNA damage repair systems could be an effective strategy to eradicate the persister population.

### Mtb Biofilm Formation Contributes to Antibiotic Tolerance

Biofilm is a three-dimensionally organized multicellular bacterial community that grows on surfaces *in vitro* and *in vivo* (70). Biofilm induces persistent bacterial infection by protecting bacteria from antibiotics (71). Therefore, the formation of biofilms has been closely linked to antibiotic tolerance in various bacterial pathogens, including *E. coli*, *S. aureus*, *P. aeruginosa*, and Mtb (72–76). Host-mediated stress conditions, such as prolonged hypoxia, oxidative stress, and nutrient starvation, induce biofilm formation leading to the development of antibiotic tolerance (77–81).

Ackart et al. showed that human leukocyte lysis enhanced biofilm formation, subsequently inducing antibiotic tolerance to several anti-TB drugs, such as INH, RIF, and pyrazinamide (PZA) (77). Interestingly, treatment with DNase I or tween scattered the established biofilm. It reversed the antibiotic tolerance, indicating that biofilm formation induced by host-mediated stress provides antibiotic tolerance that leads to persistent infection, and targeting the biofilm enhances drug sensitivity in Mtb (77). Another study induced Mtb biofilm formation *in vitro* through thiol reductive stress (TRS), resulting in drug-tolerant (INH, RIF, and EMB) phenotypes in which metabolic activity was maintained with the same levels of ATP/ADP, NAD+/NADH, and NADP+/NADPH (78). Furthermore, the TRS-induced biofilm formation was not interrupted by cell wall biosynthesis inhibitor antibiotics (INH and ETB), while DNA synthesis (levofloxacin and ofloxacin), RNA transcription (RIF), and protein synthesis (tetracycline) inhibitors disrupted its formation (78). Recently, Richards et al. identified several indispensable genes for Mtb adaptation during biofilm formation induced by host-mediated stresses, but not in dispersed culture using detergent (79). They observed that the formation of biofilm enhances the enrichment of antibiotic-tolerant cells and subsequently inducing RIF tolerance. Importantly, they established that isonitrile lipopeptide is essential for the structural formation of Mtb biofilm under stress conditions (79). In another study, modulation of trehalose metabolism was observed in antibiotic-tolerant Mtb population isolated from a biofilm; antibiotic-tolerant Mtb utilize trehalose to synthesize central carbon metabolism intermediates required to sustain mandatory cellular functions, whereas planktonic cells use cell-surface glycolipids (81). Moreover, drug-susceptible and MDR Mtb showed similar alteration after antibiotic therapy, suggesting the role of trehalose in both transient and permanent antibiotic tolerance (81). Tripathi et al. showed that ClpB is essential for Mtb survival under host-mediated stress conditions; they demonstrated that ClpB is required for bacterial survival during hypoxia and nutrient starvation (80). The clpB deletion mutant showed abnormal cellular morphology, disrupted biofilm formation, and reduced rate of intracellular survival in THP-1 cells (80). In addition, they showed that ClpB induces the secretion of inflammatory cytokines, such as TNF-α and IL-6, controlled by MAPK and NF-κB pathways (80). Taken together, various virulence factors involved in Mtb biofilm formation may be a potential novel drug target for the elimination of drug-tolerant bacteria.

## Antibiotics Can Affect Host Immunity and Influence Clinical Outcomes

### Host Metabolic Changes Induced by Antibiotics

Antibiotic activities against bacterial pathogens have traditionally been considered only in terms of their direct killing effects (82). However, growing evidence indicates indirect effects of antibiotics through interaction with host innate immunity that can alter clinical outcomes (83–85). Yang et al. identified antibiotic-induced host metabolic changes during infection and found that antibiotic treatment directly induced the host cells to produce metabolites that reduce drug efficacy and amplify phagocytic killing (86). After ciprofloxacin treatment, the systemic alteration of metabolites was confirmed in mouse tissues, including the peritoneum, plasma, and lungs. On the contrary, *E. coli* infection induced local changes in the peritoneum alone, not in the plasma or lungs (86). Further, most of the antibiotic-induced metabolic changes were not reliant on the intestinal microbiome and were most likely caused by the direct action of antibiotics on local host cells (86).

Above all, advanced host types of machinery protect cells by detecting and preventing damage due to intrinsic and exogenous inferior substances, such as oxidative stress and toxins (87). These apparatuses are partially responsible for microbial pathogenesis by detecting endogenous factors induced by Mtb infection or exogenous Mtb factors; however, they are responsible for detecting and detoxifying anti-TB drugs or drug-induced endogenous factors (88). These apparatuses may belong to the NRF2-KEAP1 and aryl hydrocarbon receptor (AhR) signaling pathways, whose dual action may be a double-edged sword in Mtb infection. This section evaluates the effect of such a system on Mtb infection and anti-TB treatment.

#### Keap1-Nrf2 Signaling Pathway

**Figure 2:**
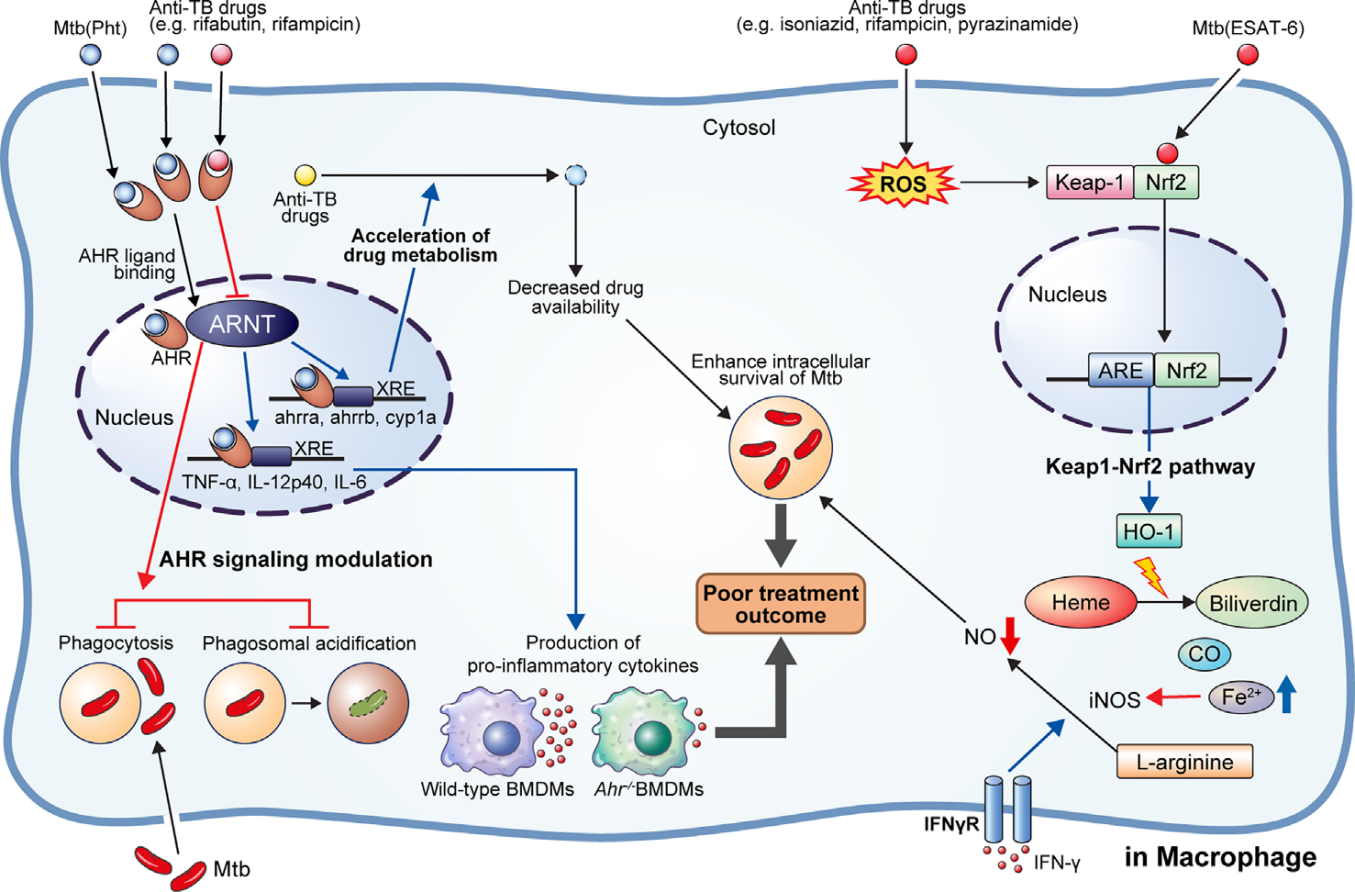
AhR modulation by anti-TB drugs and downstream events. NRF2-KEAP1 signaling and AhR signaling pathways generally protect cells by detecting and preventing damage to endogenous and exogenous substances such as oxidative stress and toxins. They can also detect *M. tuberculosis* (Mtb) infection or anti-TB drugs and affect host defense and drug metabolism. 1,4-naphthoquinone phthiochol (Pht) produced by Mtb and anti-TB drugs can attach to AhR present in the cytoplasm across the cell membrane. The combined ligand and receptor complex transfers into the nucleus and heterodimerizes with AhR nuclear translocator (ARNT). The ligand, receptor, and ARNT complex binds to xenobiotic response elements (XRE) that are specific DNA sequences found in the target gene promoter region. Activation of the AhR by Pht and anti-TB drugs (e.g., rifabutin, bedaquiline) in macrophages induces impaired phagocytosis of Mtb H37Rv, and phagosome acidification, and production of pro-inflammatory cytokines. Furthermore, activation of AhR facilitates the hepatic metabolism of drugs, ultimately reducing drug availability. Meanwhile, some drugs (e.g., rifampicin) act as AhR inhibitors, inducing impairment of phagocytosis and phagosome acidification, consequently improving the intracellular survival of Mtb in macrophage and zebrafish models. On the other hand, Mtb ESAT-6 and anti-tuberculosis drugs (e.g., isoniazid, rifampicin, pyrazinamide) act on Nrf2-Keap1 signaling to induce the translocation of Nrf2 to the nucleus by degradation of Keap-1. The translocated Nrf2 binds to the antioxidant response element (ARE) and upregulates antioxidant enzymes. Production of hemeoxygenase-1 (HO-1), a representative antioxidant enzyme, is activated, which subsequently induces catabolism of heme to biliverdin, CO, and Fe^2+^. Elevated Fe^2+^ inhibits the production of nitric oxide from L-arginine mediated by IFN-γ signaling. Thus, activation of heme catabolism by HO-1 induces the reduction of intracellular bacterial killing. Taken together, activation of AhR signaling and HO-1 production induces a pathogen-beneficial effect that enables persistent infection.

The Keap1-Nrf2 regulatory pathway is a key mechanism for preventing cell damage by detecting intrinsic and exogenous stresses, such as oxidative stress, chemotherapy, and radiation, regulating gene expression to modulate various subsequent antioxidant functions (87). Nrf2 is bound to the inhibitory protein Keap1 in the cytoplasm. When a stressor is detected, the Nrf2 protein is separated from Keap1, causing its cytoplasmic accumulation. Thereafter, it translocates to the cell nucleus, where it acts as a transcription factor, binds to the antioxidant reaction factor (ARE), and then binds to the antioxidant-related genes [e.g., hemeoxygenase-1 (HO-1), NAD(P)H:quinone redox enzyme-1 (NQO1), glutathione S-transferase (GST)] to promote their transcriptional expression (**Figure 2**) (87). Mtb factors, such as ESAT-6, can induce oxidative damage and apoptosis, counteracted by upregulating antioxidant enzymes *via* activation of the Keap1-Nrf2 signaling cascade (88, 89). Recent studies have shown that the antioxidant factor expressed by activation of the Keap1-Nrf2 system protects cells by removing infection and damage caused by drugs; however, it also inhibits T cell activation and rather hinders the removal of Mtb. Representatively, HO-1 is a cellular antioxidant enzyme expressed in response to various stress conditions, such as exposure to heavy metals, heat shock, hypoxia, starvation, and immune activation (90–95). HO-1 is the rate-limiting enzyme that degrades heme molecules into free iron, biliverdin, and carbon monoxide (CO) (96). Free iron inhibits nitric oxide (NO) production by acting on inducible NO synthase (iNOS) and, thereby, could improve the survival of intracellular Mtb.

In addition, bactericidal antibiotics cause mitochondrial dysfunction and oxidative damage in mammalian cells (97). A dose- and time-dependent upregulation of intracellular ROS production was confirmed in different human cell lines after treatment with bactericidal antibiotics (ciprofloxacin, ampicillin, and kanamycin) belonging to different classes. Moreover, mitochondrial potential, ATP levels, and metabolic activity were considerably decreased after this treatment, suggesting impairment of mitochondrial function (97). Furthermore, treating human sinonasal epithelial cells with the bactericidal antibiotics, amoxicillin and levofloxacin, leads to increased ROS production, antioxidant gene expression, and cell death (98).

Bactericidal anti-TB drugs, such as RIF, INH, and PZA, can similarly cause mitochondrial dysfunction and oxidative damage in host cells, leading to apoptosis, in addition to their effect on Mtb. Simultaneously, antioxidant mechanisms, such as the Keap1-Nrf2 signaling pathway, may interfere with the removal of Mtb. Interestingly, ROS-mediated damage induced by antibiotics could be rescued by N-acetyl-l-cysteine (NAC) without affecting the antibiotic’s killing ability (97). In addition, an HO-1 inhibitor showed the same effect in the lungs of Mtb-infected mice as anti-TB drugs (99). Thus, the long-term use of bactericidal anti-TB drugs can induce cell death due to ROS production and mitochondrial dysfunction, while simultaneously, the produced ROS act as antioxidants and interfere with the removal of Mtb. These ambivalences need to be more clearly elucidated with respect to the pathogenesis of Mtb. The aforementioned adjuvant treatments are described in detail in section 4.

#### AhR Signaling Pathway

The AhR is a transcription factor that detects both endogenous and exogenous ligands (100). Initially, AhR function was associated with the detoxification of heterologous ligands, such as benzo[a]pyrene and the highly toxic 2,3,7,8-tetrachlorodibenzo-p-dioxin (TCDD); subsequently, endogenous molecules, such as tryptophan (Trp), kynurenine, or formindolo [3,2-b] carbazole (FICZ), dietary components, and bacterial-derived ligands, were identified as AhR ligands, broadening the understanding of their function (100).

Particularly, bacterial pigment proteins such as phenazine produced by *P. aeruginosa* and 1,4-naphthoquinone phthiochol (Pht) produced by Mtb have been identified as bacterial-derived AhR ligands (101). AhR is widely expressed in almost all cell types; in particular, both innate and adaptive immune cells express AhR, suggesting its potential broad-range effects on host immunity (102). AhR is present in the cytoplasm and is activated upon binding to a ligand. Activated AhR binds to the AhR nuclear translocator (ARNT) and regulates the transcription of several target genes, including cytochrome P450 monooxygenases (*CYP1A1* and *CYP1B1*), AhR inhibitor, and pro-inflammatory cytokines (102) (**Figure 2**).

Ligand-activated AhR translocates to the nucleus from the cytosol and induces immunosuppressive or pro-inflammatory downstream effects depending on the ligand property (102). Further, AhR can modulate macrophage immune response. Shinde et al. showed that phagocytosis of apoptotic cells through toll-like receptor (TLR)9-dependent sensing of the apoptotic cell DNA induces the activation of the AhR pathway (103).

The ligand-activated AhR binds to TB virulence factors and regulates antibacterial responses (104). Puyskens et al. demonstrated that anti-TB drugs, such as RIF and rifabutin bind to AhR and induce modulation of host immune response (104). However, AhR signaling inhibition by a synthetic AhR inhibitor, CH-223191, impairs phagocytosis in THP-1 macrophages. Further, they demonstrated that the rate of internalized zymosan was decreased following RIF treatment, while phagosome acidification was also impaired after RIF as well as CH-223191 treatment (104). In addition to this *in vitro* study, they confirmed AhR modulation during *M. marinum* infection in a zebrafish model; a higher bacterial burden was observed in zebrafish embryo following AhR inhibition with CH-223191 than in the untreated control group (104). Similarly, Moura-Alves et al. demonstrated significantly increased bacterial burden in the lungs, liver, and spleen of *AhR* ^-/-^ mice than wild type mice after aerosol Mtb infection (101). Moreover, the production of pro-inflammatory cytokines, such as TNF-α, IL-12p40, and IL-6, were hindered in *AhR* ^-/-^ bone marrow-derived macrophages (101). Furthermore, Memari et al. demonstrated that AhR induced expression of IL-23 and IL-1β, thereby stimulating the production of IL-17 and 22 by specific T cell subsets (Th17, Th22, and ILC3 cells) (105). Upregulation of IL-17 activates parenchymal cells and subsequently induces an influx of polymorphonuclear cells to the infection site mediated by CXCL1, CXCL3, and CXCL5 (106). Further, phagocytosis of apoptotic polymorphonuclear cells by macrophages promotes a phenotypic change of macrophage from M0 to M2c, thereby contributing to inflammation resolution (106).

### Modulation of Host Immunity by Anti-TB Drugs

Antibiotics can modulate host immunity either indirectly or directly (107). First, antibiotics alter the host immune system indirectly by affecting the host microbiota composition (108). Second, antibiotics affect the host immune system directly by altering the functions of immune cells (86, 97, 109). Therefore, the interaction between antibiotics and host immunity may influence the clinical outcomes or treatment duration. Several studies have reported the modulation of host immune response by anti-TB drugs. For example, INH induces the apoptosis of activated CD4^+^ T cells in Mtb-infected mice (110) as well as impairs the production of Mtb-specific interferon (IFN)-γ and anti-CFP10 antibody in household contacts of latent TB patients (111). Similarly, RIF reportedly exerts a mild immunosuppression effect, as indicated by its inhibition of human lymphocytes (112) and significant suppression of T cells compared to that in TB patients without RIF treatment (113). Moreover, RIF partially suppressed the phagocytosis of zymosan by macrophages and moderately suppressed the expression of TNF-α at high doses (114). it was reported to significantly inhibit the secretion of IL-1β and TNF-α while increasing the secretion of IL-6 and IL-10 (115). Furthermore, RIF suppressed LPS-induced production of iNOs, cyclooxygenase-2, IL-1β, TNF-α, and prostaglandin E2 in microglial cells, subsequently improving neuron survival (116). Manca et al. demonstrated that PZA treatment reduces the secretion of pro-inflammatory cytokines and chemokines, such as IL-1β, IL-6, TNF-α, and MCP-1, in Mtb-infected human monocytes and mice (117). Additionally, PZA treatment elevated the expression of adenylate cyclase and peroxisome-proliferator activated receptor in the lungs of Mtb-infected mice (117).

Bedaquiline (BDQ) specifically disrupts intracellular ATP production in bacteria by inhibiting the activity of bacterial ATP synthase, resulting in depleted energy production (118, 119). Recently, a genome-wide transcriptional analysis demonstrated that BDQ promotes the formation of lysosomes, phagocytic vesicle membrane, vacuolar lumen, hydrolase activity, and lipid homeostasis in naïve and Mtb-infected macrophages (120). Moreover, it suppressed basal glycolysis, reduced glycolytic capacity in heat-killed-Mtb-stimulated macrophages, and triggered anti-mycobacterial mechanisms, such as phagosome–lysosome fusion and autophagy (120). Further, BDQ treatment induced the activation of the lysosomal pathway through transcription factor EB and calcium signaling. Interestingly, other classical anti-TB drugs, such as amikacin, EMB, and INH, did not activate the lysosomal pathway. Additionally, BDQ potentiated the anti-mycobacterial activity of PZA but did not show synergistic effects with bactericidal activities of EMB, INH, and RIF (120).

Clofazimine (CFZ) is a riminophenazine compound used for the standard treatment of leprosy (121). In addition to its antimicrobial activity, CFZ has an immune-modulatory activity; CFZ forms biocrystal and modulates innate immune response after phagocytosis as demonstrated by the intracellular CFZ crystal-induced activation of the Akt pathway and enhancement of IL-1RA production in RAW 264.7 cells (122). Moreover, CFZ treatment inhibited TLR2-and TLR4-mediated NF-κB activation and TNF-α production (122). Fukutomi et al. demonstrated that CFZ induces apoptosis of macrophages; representative features of apoptosis, such as decreased metabolic activity, diminished cell size, nuclear condensation, and fragmentation, were observed in CFZ-treated human monocyte-derived macrophages (123). Further, caspase-3 activity was significantly increased in CFZ-treated macrophages (123). Recently, Ahmad et al. showed that BCG revaccination with CFZ treatment induces the differentiation of the stem cell-like memory T (Tsm) cells in mice (124). Differentiation of Tsm cells recovered long-lasting central memory T cells and T effector memory cells to provide enhanced vaccine efficacy in mice (124).

## Adjunctive Host-Directed Therapies Improve Anti-TB Drug Activity

Anti-TB therapy involves the combination of several drugs and has a long treatment duration of treatment, resulting in the frequent occurrence of side effects (125, 126). Side effects range from minor ones that disappear spontaneously to serious ones that require treatment (126). The strategies for developing new treatments to control Mtb can be divided into two broad categories: developing novel efficient antibiotics and using existing therapeutic drugs to achieve faster and more effective treatments in a host-specific manner (127, 128). The development of host-directed therapy maximizes treatment efficiency by using the adjunct to elicit a response (127). Essentially, these treatments do not directly target pathogens, thus avoiding the occurrence of drug resistance and reducing drug side effects, thereby making this a promising strategy (127). In this section, we have proposed several such strategies, including various host targets that affect Mtb susceptibility, and discussed the corresponding drugs and their mechanisms of action. A summary of the proposed adjunctive host-directed therapies that improving anti-TB drug activity is presented in **Table 1**.

**Table 1:** Effect of TB representative adjunctive therapeutic agents of anti-TB drugs on host immunity.

Therapeutic agent	Mechanism of action	Role in TB	Model	Therapeutic effect or outcome	References
Rapamycin	Inhibits mTOR complex	Enhances autophagy and antigen presentation	Mouse (C57BL/6)	Increased Ag85B-specific T cell responses	(129)
			Macrophage (THP-1)	Inhibition of Mtb growth	(130)
			Mouse (BALB/c)	Reduced pathological lesion and Mtb burden	(131)
Metformin	Activates the AMPK	Enhances autophagy and reduces inflammation	Mouse (C57BL/6)	Reduced pathological lesion and enhanced Th1 immune response	(132)
			Clinical trial	Decreased mortality during TB treatment in diabetes patients	(133)
			PBMCs	Lowered TNF-α, IFN-γ, and IL-1β Increased phagocytosis and ROS production	(134)
			Guinea pigs	Decreased pathological severity	(135)
Statins	Inhibits HMG-CoA reductase Increase intracellular Ca^2+^	Enhancing autophagy and phagosome maturation	MDM	Decreased intracellular Mtb survival by statin monotherapy	(136)
			Mouse (C57BL/6)	Decreased intracellular Mtb survival Decreased pathological severity	
			Macrophage (J774)	Decreased intracellular Mtb survival	(137)
			Mouse (BALB/c)	Enhanced bactericidal activity of anti-TB drugs	
			Macrophage (THP-1)	Decreased intracellular Mtb survival	(138)
			Mouse (BALB/c)	Decreased intracellular Mtb survival Reduced TB relapse rates	
			PBMCs	Decreased intracellular Mtb survival	(139)
			Macrophage (THP-1)	Reduced Mtb growth	(140)
			Mouse (C3HeB/FeJ)	Enhanced bactericidal activity of anti-TB drugs	
NAC	ROS scavenging Increase intracellular GSH	Reduces oxidative stress/inflammation	Randomized clinical trial	Reduction of anti-TB drug-induced hepatotoxicity	(141)
			Guinea pig	Decreased intracellular Mtb survival Decreased pathological severity	(142)
			Macrophage (THP-1)	Decreased intracellular Mtb survival	(143)
			Mouse (C57BL/6) (*gp91Phox* ^−/−^)	Decreased intracellular Mtb survival by NAC monotherapy	
			Randomized clinical trial	Clearing of lung infiltration Reduction of cavity size	(144)
			Human granuloma	Decreased intracellular Mtb survival Formation of solid stable granuloma	(145)
			Macrophage (THP-1)	Synergistic effect on bactericidal activity of anti-TB drugs	(146)
			Randomized clinical trial in TB/HIV co-infected patients	No significant change between NAC-treated and non-treated groups	(147)
			*In vitro*	NAC potentiates the activity of anti-TB drugs	(148)
			Macrophage (THP-1/J774)	Reduced intracellular Mtb survival in THP1, but not in J774	
			Mouse (CBA/J)	Co-treatment of NAC potentiates the activity of anti-TB drugs, but disappeared at the later time point	
Verapamil	Inhibits the calcium ion channel	Inhibits the drug efflux pump of Mtb	Mouse (C3HeB/FeJ)	Co-treatment of verapamil with anti-TB drugs significantly lowered lung bacterial loads and relapse rates compared to standard therapy alone	(149)
			Mouse (BALB/c)	Co-treatment of verapamil with a combination regimen of moxifloxacin and linezolid showed a significant reduction in lung mycobacterial load	(150)
		Disrupts membrane potential of Mtb	*In vitro*	VP kills exponentially growing, stationary-phase and nutrient-starved non-replicating Mtb	(151)
		Increase drug bioavailability and efficacy	Mouse (CD-1)	VP increases plasma concentration of RIF	(151)
			Mouse (BALB/c)	Co-treatment of BDQ with VP increased the plasma exposure for BDQ	(152)

### Autophagy-Modulating Drugs

Autophagy is an intracellular self-degradation system that transfers cytoplasmic components or specific cytosolic targets to the lysosome for cellular homeostasis maintenance (153). Autophagy is induced by various stress conditions, such as nutrient starvation, hypoxia, and microbial infection (153). Furthermore, diverse pathophysiological conditions, such as aging, autoimmune disease, neurodegeneration, cancer, and inflammation-associated metabolic disorders, are involved in autophagy (154). Autophagy is an essential part of the host immune system against diverse intracellular pathogens, such as *Salmonella*, *Listeria monocytogenes*, and Mtb, *via* the activation of phagolysosome formation (155–157). Therefore, activating autophagy is a promising strategy for eradicating Mtb, especially in the case of MDR-TB.

#### Rapamycin

**Figure 3:**
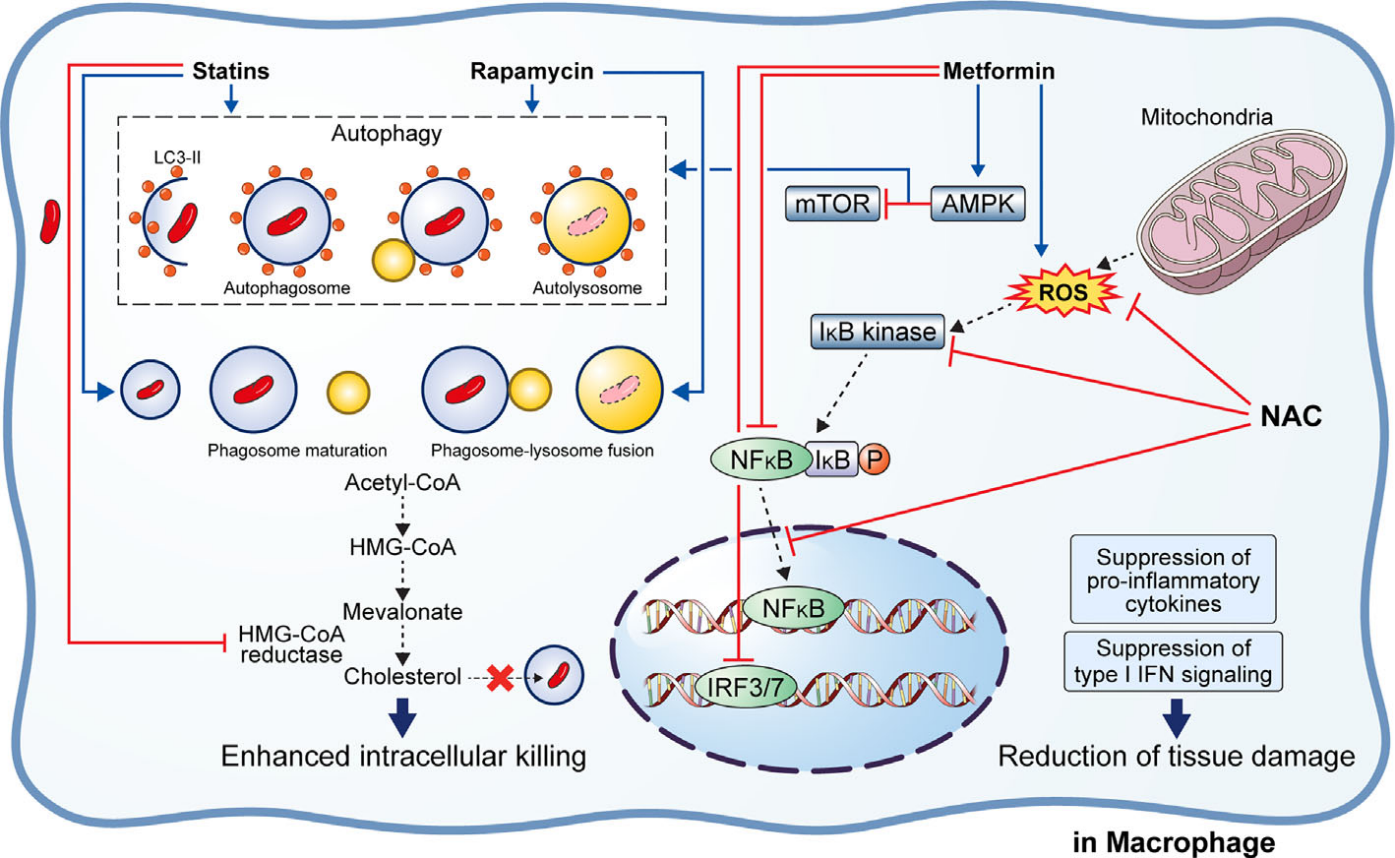
Effect of adjunctive therapeutic agents of anti-TB drugs on host immunity. Various adjunctive drugs aid TB treatment by modulating the host immune response. *M. tuberculosis* (Mtb) can accumulate cholesterol for use as a source of carbon and energy. Statins bind to the active site of HMG-CoA reductase, thereby inhibiting cholesterol biosynthesis. In addition, statins induce autophagy and phagosome maturation to promote the removal of Mtb. Similarly, rapamycin induces autophagy and phagosome–lysosome fusion to enhance the intracellular killing of Mtb. Metformin inhibits the mTOR complex via AMPK activation in the mitochondria to promote autophagy. Metformin also inhibits ROS production, NFκB signaling, and type I interferon signaling. Similarly, N-acetyl-l-cysteine eliminates the generated ROS and inhibits NFκB signaling. Suppression of pro-inflammatory immune response and type I interferon signaling lead to reduced immunopathological severity that beneficial to the host.

Rapamycin (RAP) is a potent antifungal agent produced by *Streptomyces hygroscopicus* and is used to suppress transplant rejection reactions due to its immunosuppressive property (158, 159). RAP also enhances the T helper 1-driven immune response when co-administered with the BCG vaccine (129). Further, RAP-loaded nanoparticles were efficiently phagocytosed by THP-1 macrophages, significantly reducing the intracellular Mtb load at a concentration of 100 μg/mL (130). In addition to the *in vitro* results, Gupta et al. showed that inhaled RAP particles reduced pulmonary Mtb loads as well as activated autophagy and phagosome–lysosome fusion in a mouse model (131) (**Figure 3**). Another study showed that a low dose of RAP (<10 μM) was sufficient to increase autophagy in RAW264.7 cells; however, intracellular mycobacteria killing was only observed at a high concentration of RAP (10 μM) (160). In contrast, Andersson et al. demonstrated that RAP facilitates the increase of Mtb burden in both single and human immunodeficiency virus (HIV) co-infected human monocyte-derived macrophages (161). They suggested that autophagy induction disrupts intracellular killing during a low dose infection. However, they used HIV co-infected human macrophages for their experiment that exhibit impaired immunity and, therefore, are not suitable for investigating the effect of RAP on autophagy.

#### Metformin

Metformin (MET) is the most commonly used medication for diabetes and has been suggested as an adjunctive agent for host-directed TB therapy (132). Singhal et al. discovered that the MET disrupts the intracellular Mtb growth, reduces immunopathological severity, and increases the efficacy of anti-TB drugs (132). In detail, MET treatment reduced the bacterial load (in terms of CFU) 35 days post-infection in Mtb-infected mice (132). The combination of MET and anti-TB drugs, such as INH and ethionamide, significantly decreased lung Mtb load in the mouse model, indicating synergism between MET and anti-TB drugs (132). Additionally, reduction of lung tissue pathology was confirmed in the MET-treated mice, and the number of lung CD8^+^ IFN-γ^+^ cells was increased in MET-treated mice in both unstimulated and Mtb-stimulated groups, suggesting an enhanced immune response to TB (132). Similarly, the protective effect of MET was confirmed in a chronically Mtb-infected guinea pig model in which reduced lung legions and Mtb CFU were observed in the MET-treated group compared to those in the untreated group (135). Further, the MET-treated animals showed a higher proportion of lymphocytes in the acute and subacute stages and well-encapsulated granulomas (135). MET reportedly reduces the immunopathological severity by reprogramming T cell metabolism (162, 163). MET-induced oxidative phosphorylation and glycolysis enhanced the host resistance to Mtb infection in the guinea pig model (135). Moreover, Degner et al. demonstrated that MET treatment reduces mortality during TB therapy in a retrospective cohort study in Taiwan (133). Another study reported that MET enhances anti-TB immune responses by altering host responses in humans (134). Additionally, MET induced a significant reduction in TNF-α, IL-1β, IL-6, IFN-γ, and IL-17 release in response to Mtb lysate (134). These studies indicate that MET protects against Mtb infection *via* modulation of inflammation and metabolism (133–135). Indeed, MET reduces the type I IFN response and pathological severity of TB while enhancing anti-TB immunity, such as autophagy, ROS production, and phagocytosis (132, 164, 165) (**Figure 3**). In contrast, Dutta et al. showed that MET-treated mice had similar lung Mtb CFU compared to control mice, and the percentage of mice with Mtb culture relapse was similar between the two groups (166). This result was contradictory to a previous *in vivo* study that used the same MET dose (250 mg/kg) (132). The major difference between the two studies is the co-injection of multiple anti-TB drugs (RIF, INH, PZA, and EMB) in the *in vivo* model compared with the single anti-TB drug (INH or EMB) used *in vitro* (132, 166). In addition to its antimicrobial activity, RIF promotes liver metabolism by activating hepatic cytochrome P450 enzymes, such as CYP2D6 and CYP3A4, thereby accelerating the drug metabolism and clearance by the liver (167). However, MET is not metabolized in the liver and is excreted *via* the urine in its unchanged form (168). Another potential explanation is that MET and RIF compete for the same drug target, namely the AMP-activated protein kinase (AMPK) in the liver (109, 169). Therefore, the combination with RIF reduces the effect of MET in the host.

#### Statin

Statins are anti-hyperlipidemic drugs that block 3-hydroxy-3-methylglutaryl coenzyme A reductase in the cholesterol synthesis pathway, thereby lowering the risk of stroke and cardiovascular diseases (170) (**Figure 3**). As cholesterol is an essential intracellular energy source for Mtb, elevated cholesterol level is a risk factor for TB (171). Statins also have immunomodulatory effects, such as the production of natural killer T cells, downregulation of MHC II expression, elevated secretion of IL-1β and IFN-γ, and increased caspase-1 enzyme activity, and thereby promote apoptosis and autophagy (172) (**Figure 3**). Both retrospective clinical trials and animal model studies have reported that statins are effective in the treatment and prevention of TB (136, 173, 174). Decreased bacterial burden was confirmed in peripheral blood mononuclear cells and monocyte-derived macrophages from patients with familial hypercholesterolemia during statin treatment compared to healthy donors (136). Further, statin treatment reduced the Mtb burden and histopathological severity in the lungs of Mtb-infected mice (136). Lobato et al. evaluated the effects of two statins (atorvastatin and simvastatin) alone and in combination with RIF on *M. leprae* and Mtb in THP-1 macrophages (175). Both statins showed bactericidal effects on intracellular mycobacteria 72 h post-infection and synergism with RIF at a concentration of 0.2 µM for reducing the viability of intracellular Mtb (175). Skerry et al. investigated the bactericidal activity of simvastatin alone and in combination with anti-TB drugs (RIF, INH, and PZA) in macrophages and a mouse model and found that the addition of 5 mM simvastatin significantly enhanced the bacterial killing of INH in Mtb-infected J774 macrophages (137). In contrast, the addition of 25 mg/kg simvastatin to the standard TB treatment regimen significantly reduced the lung bacterial burden in BALB/c mice (137). Similarly, Dutta et al. found that the addition of simvastatin (60 mg/kg) to the TB treatment regimen (INH/RIF/PZA) shortened the duration required to attain culture-negative results from 4.5 to 3.5 months, i.e., shortened the treatment duration (138). Simvastatin significantly improved the bactericidal activities of anti-TB drugs against intracellular Mtb while having no effect on intracellular RIF concentrations (138). The same research group further showed that various statins, including pravastatin, simvastatin, and Fluvastatin, improved the antimicrobial activity of INH, RIF, and PZA in THP-1 cells and the C3HeB/FeJ mouse model (140). Additionally, pravastatin induced phagosome–lysosome fusion and macrophage activation observed in IFN-γ- and LPS-activated macrophages (140). Interestingly, Guerra-De-Blas et al. demonstrated that simvastatin alone significantly reduced bacterial load in Mtb-infected PBMCs *via* enhanced production of natural killer T cells, upregulation of co-stimulatory molecules in monocytes, increased the secretion of IL-1β and IL-12p70, and promotion of apoptosis and autophagy in monocytes (139).

### ROS Modulating Drugs

ROS and reactive nitrogen species (RNS) are critical host defense mechanisms to eradicate pathogens during infection (176). The ROS and RNS react with the phagosomes and efficiently eliminate intracellular bacteria. However, excessive ROS production may cause mitochondrial damage and cell apoptosis, leading to severe immunopathologic outcomes (177). Therefore, appropriately balanced cellular ROS levels are critical for eliminating intracellular Mtb without causing a detrimental effect in the host.

#### N-Acetyl-Cysteine (NAC)

Multiple studies have shown that reducing ROS accumulation in the Mtb-infected host by NAC inhibited the Mtb growth and reduced the immunopathological severity despite the contradictory views on the Mtb killing ability of NAC (142, 143, 145, 148, 178–182) (**Figure 3**). Several studies have demonstrated that NAC ameliorates aminoglycoside-induced ototoxicity (183–185). Venketaraman et al. found that glutathione level was significantly reduced in PBMCs and RBCs isolated from TB patients (181). Further, reduced secretion of IL-10, IL-6, TNF-α, and IL-1 was confirmed in blood cultures of TB patients after NAC treatment (181). Similarly, Palanisamy et al. showed that NAC treatment moderately increased blood glutathione level and the serum antioxidant capacity in Mtb-infected guinea pigs, reduced the bacterial burden in the spleen, and decreased the immunopathological severity in the lungs and spleen of animals (142). Subsequently, Guerra et al. demonstrated that increased glutathione level due to NAC treatment inhibits the intracellular growth of Mtb by causing an increase in the levels of IL-2, IL-12, and IFN-γ secreted by T cells (180). Furthermore, NAC attenuates liver injury induced by anti-TB drugs by promoting free radical scavenging and glutathione synthesis (141).

Similarly, Amaral et al. demonstrated that NAC inhibits the growth of diverse pathogenic mycobacteria such as Mtb, *M. bovis*, and *M. avium* (143). The mycobacterial loads in the lungs of NAC-treated animals were significantly reduced compared to that in the untreated animals in both wild type and *gp91Phox* ^-/-^ macrophages, suggesting that the anti-TB activity of NAC is independent of the host NADPH oxidase system (143). Lamprecht et al. showed that NAC potentiates the bactericidal activity of BDQ, Q203, and CFZ in an *in vitro* macrophage model (179). They found that the addition of NAC significantly improved bactericidal activity of the three anti-TB drugs, leading to complete Mtb sterilization (179). In another study, NAC treatment caused a 50% reduction in bacterial load (in terms of CFU) in THP-1 macrophages and potentiated the bactericidal effect of anti-TB drugs, such as INH, RIF, EMB, or PZA (146). Moreover, NAC treatment can modulate TNF-α levels to maintain granuloma structure without inducing detrimental cell damage to the host (146). Similarly, Teskey et al. showed that incubation of Mtb Erdman strain with NAC significantly inhibited the bacterial growth, while incubation with a combination of NAC and anti-TB drugs (INH, RIF, and EMB) completely sterilized the Mtb culture (145). In addition, NAC treatment significantly increased the IFN-γ level while decreasing that of TNF-α as well as significantly enhanced the phagosome acidification in human granulomas, which indicates improved intracellular killing (145). On the contrary, NAC alone did not kill Mtb in macrophages, whereas INH and NAC combined showed an improved bactericidal activity than INH alone (178). However, Khameneh et al. demonstrated that the combination of anti-TB drugs (RIF and INH) and vitamin C, but not NAC, induced synergistic effects for bacterial killing (182). However, there were a few inconsistencies in their results. For instance, in the presence of 20 μg/mL RIF, treatment with 0.05 mg/mL NAC showed 10% CFU, whereas treatment with 0.1 mg/mL NAC showed 150% CFU compared to untreated controls (182). Recently, Vilchèze et al. showed that NAC improves the sterilizing activity of first and second-line anti-TB drugs *in vitro* against drug-susceptible and drug-resistant Mtb strains (148). However, a synergistic effect between NAC and anti-TB drugs was not observed in Mtb-infected mice (148). Moreover, although NAC initially inhibited Mtb growth, the NAC-induced growth inhibition was not significant and was lost after the first week of treatment (148). The major difference between the controversial studies is the host species. The direct killing effect of NAC was not seen in studies using *in vivo* or *in vitro* mouse models. Similarly, clinical trials on the adjunct effect of NAC on TB therapy in TB patients showed contradictory results (144, 147). Mahakalkar et al. showed that NAC treatment significantly shortened the duration of anti-TB therapy in TB patients (144). On the contrary, a recent clinical trial in Brazil demonstrated that NAC addition to a standard TB regimen did not reduce the duration required to achieve a negative sputum culture, nor did it reduce radiological severity in hospitalized patients with severe TB and HIV co-infection (147) (**Table 1**). However, these trials did not include a sufficiently large study population. Therefore, a large-scale clinical study is needed to determine the safety and efficacy of NAC treatment in TB.

#### Vitamin C

Vitamin C (VC) is an essential nutrient for humans that possesses reducing and antioxidant abilities associated with its ability to donate electrons (186). Several studies have investigated the host beneficial or detrimental roles of VC in the pathogenesis of TB (187–192). Vilchèze et al. demonstrated that VC kills drug-susceptible and drug-resistant Mtb *via* Fenton reaction in a dose-dependent manner *in vitro*, and 4 mM of VC completely sterilized Mtb culture at three weeks after treatment (187). VC is assumed to kill Mtb by increasing the intracellular ROS level, and this process depends on the intracellular iron concentration (187). The same research group also showed that a combination of VC and anti-TB drugs sterilizes Mtb cultures faster than monotherapy with anti-TB drugs (190). Further, Susanto et al. revealed that administration of VC improves the sputum conversion culture rate in RIF-susceptible Mtb-infected patients (189). On the contrary, Sikri et al. suggested that VC induces Mtb dormancy leading to a viable but non-culturable state (188). VC-treated Mtb showed antibiotic tolerance, thereby exhibiting a higher survival rate than untreated Mtb culture in the presence of anti-TB drugs (188). Similarly, Nandi et al. demonstrated that VC induces the activation of multiple transcriptional regulators for the temporal adaptation to VC, leading to a dormancy response (191). In summary, VC sterilizes Mtb culture by generating ROS *via* Fenton reaction and promoting oxygen consumption, thus eradicating bacterial persisters. However, several studies report that VC promotes the generation of bacterial persisters in TB. Therefore, further research is needed to elucidate the role of VC in the pathogenesis of TB.

### HO-1 Inhibitor

In section 3, we discussed that Mtb and bactericidal anti-TB drugs cause mitochondrial dysfunction and oxidative damage in host cells, consequently ROS-mediated apoptosis as well as simultaneous activation of antioxidant mechanisms, such as the Keap1-Nrf2 signaling pathway, that may interfere with the removal of Mtb (88, 89) A major factor involved in this mechanism is HO-1 (95, 192). However, the detailed role of host HO-1 during the onset and pathogenesis of TB remains controversial and has not been fully elucidated (95, 99, 192–195). HO-1 exerts anti-inflammatory and cytoprotective effects, although the underlying mechanisms are not fully understood (196). Several studies have investigated the host beneficial or detrimental roles of HO-1 during TB infection (95, 99, 192–195). Andrade et al. showed that active TB patients show a negative correlation between plasma levels of HO-1 and MMP-1 (95). Notably, the TB patients with high plasma levels of HO-1 or MMP-1 demonstrated unique clinical presentation and inflammatory cytokine profiles (95). Moreover, a high HO-1 level was induced by the infection of virulent Mtb strain in human or murine macrophages, and MMP-1 expression was inhibited by CO by suppressing c-Jun/AP-1 signaling (95). Costa and colleagues demonstrated that the administration of tin protoporphyrin IX (SnPPIX), an HO-1 enzymatic inhibitor, decreases pulmonary Mtb loads comparable to that accomplished by anti-TB drug therapy as well as improves the bactericidal activity of anti-TB drugs (RIF, INH, and PZA) (192). Interestingly, host T cell immune response was needed to inhibit HO-1 by SnPPIX, and SnPPIX failed to reduce bacterial growth and activity of Mtb HO-1 enzyme in broth culture (192). Rockwood et al. reported HO-1 upregulation in Mtb-infected rabbits, mice, and non-human primates, and anti-TB therapy reduced the HO-1 plasma levels (193). Similar upregulation of HO-1 was observed in the plasma of untreated HIV-1 co-infected TB patients. In these patients, the plasma HO-1 levels positively and negatively correlated with the HIV-1 viral load and CD4^+^ T cell count, respectively (193). Further, early secreted antigen ESAT-6-mediated nuclear translocation of transcription factor NRF-2 is required for Mtb-induced HO-1 expression (193). Recently, Costa et al. discovered that HO-1 inhibition improves IFN-γ-induced NOS2-dependent bacterial killing by murine macrophages (99). Additionally, HO-1 inhibition induced low intracellular non-protein bound iron in Mtb-infected macrophages and reduced iron deposition in the lungs of Mtb-infected mice (99). Taken together, HO-1 expression inhibits T cell-mediated IFN-γ-induced NOS2-dependent control of Mtb by producing free iron. Therefore, inhibition of HO-1 expression potentiates the anti-TB therapy and improves clinical outcomes.

### Calcium Channel Blocker

Verapamil (VP) is a calcium channel blocker used for hypertension treatment that also acts as an inhibitor of drug efflux protein. Several studies have proposed that VP can potentially improve the bactericidal activity of anti-TB drugs, such as RIF, INH, EMB, BDQ, and CFZ (151, 197–199). Machado et al. demonstrated that VP disrupts the heightened antibiotic resistance induced by repetitive exposure to INH (197). Similarly, Gupta et al. showed that VP potentiates the bactericidal activity of BDQ in reference strain H37Rv and eight clinical Mtb isolates (198). Similarly, Li et al. suggested that the addition of VP improves RIF susceptibility in RIF-resistant Mtb isolates (199). Chen et al. suggested that VP monotherapy kills exponentially growing, stationary-phase, nutrient-starved, non-replicating Mtb by disrupting membrane energetics without affecting the physical integrity of the membrane (151). Further, VP potentiates the bactericidal effects of BDQ and CFZ *in vitro* and in a RIF mouse model without changing the intracellular concentration of the drugs (151). Similarly, Xu et al. demonstrated that VP potentiates the efficacy of BDQ and CFZ against Mtb clinical isolates (152). However, VP increased bioavailability and efficacy of BDQ but not CFZ in Mtb-infected mice (152). Collectively, synergistic activity of VP *in vivo* may be attributed to improved systemic exposure to co-treated drugs by modulating mammalian transporters without inhibiting bacterial efflux pumps (152). Thus, the combination of VP and anti-TB drugs may be an effective therapy for TB.

### Cytokines

Cytokines are small soluble proteins with multifaced aspects of host protective and detrimental effects (200). Excessive TB-induced inflammation interferes with normal lung function and may increase the risk of TB relapse (201). Comprehensive insight into host-pathogen interaction in TB would aid in designing host-directed therapies to shorten antibiotic treatment duration and relieve immunopathology. Immunotherapy with several cytokines protects the host and boosts bacterial clearance (**Table 2**).

**Table 2:** Effect of cytokines on host immunity in TB.

Cytokine	Role in TB	Model	Therapeutic effect or outcome	References
GM-CSF	Restriction of Mtb burden Lymphocyte recruitment Formation of normal granulomas	Mouse (C57BL/6)	Prevented weight loss and enhanced pulmonary Mtb clearance	(202)
		Mouse (BALB/c)	Exogenous administration GM-CSF induced significant reduction of pulmonary bacterial loads	(203)
		Mouse (BALB/c)	Exogenous administration GM-CSF induced significant reduction of pulmonary bacterial loads and pneumonic area	(204)
		Mouse (C57BL/6)	GM-CSF neutralization reduces acute lung inflammation and neutrophil recruitment	(205)
		Mouse (C57BL/6)	GM-CSF neutralization induces increased pathological lesion, necrosis, inflammation, and pulmonary Mtb burden	(206)
IFN-γ	Mediator of macrophage activation	Randomized clinical trial	Increased rate of Mtb clearance Significant reduction of prevalence of clinical symptoms such as fever, sneeze, and night sweats	(207)
		Mouse (BALB/c)	Exogenous administration of IFN-γ reduced bacterial loads and tissue damage in the lung	(208)
		Macrophage (MDM)	Pretreatment of IFN-γ impaired immune response of MDM from MDR-TB patients	(209)
Type I interferons (IFN-α/IFN-β)	Suppression of pro-inflammatory cytokines and Th1 responses	Mouse (C57BL/6)	Overexpression of type I interferons induced increased pulmonary Mtb loads	(210)
		Mouse (C57BL/6)	*Ifnar^-/-^*/*Ifngr^-/-^* mice showed decreased survival rate and increased Mtb loads in the lung	(211)
		Mouse (C57BL/6)	*Ifnar* ^-/-^ mice showed similar Mtb loads in the lung	(206)
		Mouse (129S2)	Suppression of type I IFN signaling significantly enhanced the bactericidal activity of RIF which leading to reduced bacterial loads and improved survival	(212)
		Mouse (C57BL/6)	*Il1r1^-/-^* mice showed decreased survival rate and increased pulmonary Mtb loads	(213)
TNF-α	Macrophage activation, critical for granuloma formation and maintenance	Analysis of reports	Infliximab therapy induced the reactivation of latent tuberculosis	(214)
		Mouse (B6D2F1)	Exogenous administration of TNF-α induced significant reduction of bacterial load and pneumonic area	(215)
		3D cell culture model	TNF-α neutralization reverses augmented Mtb growth caused by anti-PD-1 treatment	(216)
		*In vitro* granuloma model	TNF-α antagonists induced resuscitation of dormant Mtb	(217)
IL-2	Promotes the expansion of the antigen-specific T cells	Clinical trials	Exogenous administration of IL-2 reduced bacterial loads in sputum	(218)
		Mouse (C57BL/6)	Exogenous administration of IL-2 restored T cell dysfunction induced by persistent Mtb infection	(219)
IL-12	Proliferation and activation of T lymphocytes, NK cells, and NKT cells	Mouse (C57BL/6)	IL-12 improved survival and reduced bacterial loads of Mtb-infected *CD4* ^-/-^ mice	(220)
		Mouse (BALB/c)	IL-12 reduced bacterial loads and immunopathological severity	(221)
IL-22	Production of inflammatory mediators and recruitment of pathologic effector cells	Mouse (C57BL/6)	*Il22^-/-^* mice showed increased bacterial loads in the lung and spleen	(222)
		Macrophage (MDM)	Exogenous administration of IL-22 induced significant reduction of intracellular growth of Mtb	(223)
IL-17	Affect neutrophil homeostasis and survival	Mouse (C57BL/6)	*Il17^-/-^* mice showed increased lung bacterial burden compared to wild type	(224)
IL-23	Induces the IFN-γ and IL-17 response in the lung and enhances host protection	Mouse (C57BL/6)	*Il23^-/-^* mice showed moderately enhanced immunopathological response in the lung	(225)
		Mouse (C57BL/6)	Exogenous administration of IL-23 significantly reduced the pulmonary Mtb loads, and the lung inflammation levels	(226)
IL-24	Induces IFN-γ production by CD8^+^ T cells	Mouse (BALB/c)	Exogenous administration of IL-24 significantly reduced the Mtb loads in the lung and spleen. Also, survival was improved in IL-24 treated group	(227)

#### IFN-γ and IL-12

IL-12/IFN-γ axis plays a critical role in host immune response for controlling TB, and IFN-γ is the key cytokine in the innate immune response during Mtb infection (219). IFN-γ is responsible for enhancing bactericidal activity through the upregulation of ROS, RNI, and autophagy (155, 228, 229). IFN-γ reverses the blockade of phagosome–lysosome fusion caused by Mtb (230). Moreover, IFN-γ stimulates the production of IL-12 in Mtb-infected macrophages, while IL-12 stimulates the production of IFN-γ by T cells and NK cells, thus activating macrophages that lead to intracellular bacterial killing (231). Dawson et al. reported a significant increase in CD4^+^ lymphocyte response and significantly reduced Mtb load in the sputum of the recombinant IFN-γ1b-treated group (207). Mata-Espinosa et al. demonstrated that exogenous administration of IFN-γ reduces bacterial loads and tissue damage in the lungs of Mtb-infected mice (208). Along similar lines, Khan et al. showed that treatment with exogenous IFN-γ restored defective immune response of MDMs isolated from MDR-TB patients (209). Exogenous administration of IL-12 improved survival and reduced the bacterial loads of Mtb-infected CD4^-/-^ mice (220). Similarly, treatment with recombinant adenovirus encoding IL-12 (AdIL‐12) significantly reduced the bacterial loads in a progressive pulmonary TB mouse model (221). AdIL-12-treated mice showed significantly higher levels of IFN-γ, TNF-α, and iNOS compared with the untreated group. In addition, AdIL-12-treated mice showed less severe pathological legions than the untreated mice (221).

#### TNF-α

TNF-α plays an important role in controlling TB in both the initial and late stages (232). TNF-α activates macrophages and contributes to the formation and maintenance of granulomas to suppress the dissemination of Mtb; however, it also induces tissue damage due to the excessive immune responses (233). Keane et al. demonstrated that treatment with TNF-α inhibitor induces the reactivation of latent TB (234). Similarly, treatment with several TNF-α antagonists differentially induced resuscitation of dormant Mtb in a 3D microgranuloma model (214). Adalimumab, a TNF-α antagonist, showed a greater resuscitation rate than etanercept through the TGF-β1-dependent pathway (214). Furthermore, exogenous administration of TNF-α significantly decreased the bacterial load and pneumonic area in Mtb-infected mice (217). In contrast, Tereza et al. demonstrated that excessive TNF-α secretion *via* PD-1 inhibition facilitated Mtb growth in a micro granuloma model (215).

#### IL-24

IL-24 plays an immune-regulatory role to induce Th1 cytokines, such as IFN-γ, IL-6, and TNF-α, during TB infection (216). Upregulated IL-24 expression was observed in BCG-vaccinated newborns, suggesting the host protective role of IL-24 during TB infection (235). Wu et al. demonstrated that administration of exogenous IL-24 induces IFN-γ upregulation, whereas IL-24 neutralization causes IFN-γ downregulation (216). Similarly, IL-24 stimulation results in the upregulation of IFN-γ-inducing cytokines, such as IL-12, IL-23, and IL-27 (216). Furthermore, Ma et al. demonstrated that administration of IL-24 induced IFN-γ production and activated CD8^+^ T cells in mice, indicating its host protective effect in TB (227). Huang et al. found that IL-24 family cytokines, such as IL-19, IL-20, and IL-22, are elevated in BCG-vaccinated non-human primates as well (236). Treatment with exogenous IL-24 significantly increased the survival rate and significantly reduced the bacterial burden compared to the control group (**Table 2**).

#### IL-2

IL-2 plays a critical regulatory role during T cell differentiation *via* induction of the transcription factor eomesodermin and perforin (237). During chronic viral infection, the production of memory T cells and memory T cell-associated molecules, such as CD127 and CD44, was observed after stimulation with low-dose IL-2 (238). IL-2 administration is proven to exert host protective effects in TB infection; it reduced or cleared bacterial burden and increased the number of CD25^+^ and CD56^+^ cells (239). A similar protective effect of exogenous IL-2 was reported by Shen et al., who showed that the IL-2-treated group showed decreased sputum smear-positive rates, whereas the control group showed increased sputum smear-positive rates (218). The IL-2-treated group also demonstrated less severe legions than the control group during TB treatment (218). Liu et al. showed that persistent stimulation with Mtb antigen induces disrupted cytokine production by T cells, and IL-2 restores the T cell dysfunction (219). Their findings suggested that administration of exogenous IL-2 leads to maintenance of immune homeostasis in the bone marrow and reactivation of the disrupted hematopoiesis by persistent Mtb infection (219).

#### Granulocyte-Macrophage Colony-Stimulating Factor (GM-CSF)

Previous studies showed that GM-CSF exerts a host protective role during TB infection (202–205, 240–242). It mediates the formation of granuloma and promotes bacterial clearance in the host (241), and induces classical activation of macrophage to M1 polarization state (242).

Treatment with recombinant adenoviruses encoding GM-CSF significantly reduced the bacterial burden in the lungs, increased the number of activated DCs, and elevated the levels of TNF-α, IFN-γ, and iNOS (203). The same group further demonstrated that exogenous administration of GM-CSF significantly reduced the pulmonary Mtb loads and pneumonic area in Mtb-infected mice (202). Similarly, Pasula et al. demonstrated that exogenous keratinocyte growth factor administration protects against Mtb infection through GM-CSF-dependent macrophage activation and phagosome–lysosome fusion (204). Moreover, neutralization of GM-CSF induced higher bacterial burden and increased immunopathologic severity with necrotic granulomatous lesions during INH/RIF treatment in TNF-α-deficient mice (205). Furthermore, GM-CSF blocking by monoclonal antibody enhanced host susceptibility and immunopathological severity in Mtb-infected mice (206). Moreover, the absence of GM-CSF signaling results in type I IFN-induced neutrophil extracellular trap formation that aggravates lung mycobacterial burden and lung pathology (206). In addition, neutrophil extracellular traps were abundant in the lung lesions of Mtb-infected C3HeB/FeJ mice and TB patients who showed impaired response to anti-TB therapy (206).

#### Type I IFNs

Type I IFNs include IFN-α, IFN-β, and several other IFNs that interact with IFNAR1 and IFNAR2 to activate a range of IFN-stimulated genes *via* STAT1 and STAT2 signaling (243, 244). Type I IFNs play a complex role in mediating beneficial and detrimental effects in the host during TB infection (244). Whole blood transcriptional profiling of active TB patients revealed that upregulation of type I IFN-αβ-inducible transcripts correlated with radiographic severity of the lungs that was restored to the level of healthy controls after anti-TB therapy (245). Recent studies have demonstrated that type I IFN-inducible blood transcriptional signature, including upregulation of *STAT1*, *IFITs*, *GBPs*, *MX1*, *OAS1*, and *IRF1*, was associated with active TB disease (246–248).

Antonelli et al. demonstrated that overexpression of type I IFNs increased pulmonary bacterial loads and extensively distributed necrotic legions in the poly-l-lysine and carboxymethylcellulose (poly-ICLC)-treated Mtb-infected mice (210). Further, a significant increase of CD11b^+^F4/80^+^Gr1int cells that showed diminished MHC II expression was confirmed in their lungs (210). Similarly, Mayer-Barber et al. showed IL-1-dependent host protection by producing eicosanoids that suppress immoderate type I IFN production and control bacterial loads (213). The *Il1r1^-/-^* mice showed a significantly decreased survival rate and increased bacterial loads in the lungs compared with the wild type mice (213) (**Table 2**). Similarly, induction of type I IFN due to loss of GM-CSF signaling or genetic susceptibility facilitated Mtb growth and increased disease severity (206). Interestingly, the same group demonstrated host protective effect of type I IFN in the lungs of Mtb-infected mice lacking IFN-γ signaling; the pulmonary bacterial loads were significantly higher in the *Ifnar^-/-^*/*Ifngr^-/-^* mice compared with that in the *Ifngr^−/−^* and *Ifnar^−/−^* mice on post-infection days 24 and 28 (211). Recently, Zhang et al. demonstrated a correlation between type I IFN signaling and cell death of Mtb-infected mouse macrophage (212). Further, suppression of type I IFN signaling significantly enhanced the bactericidal activity of RIF in Mtb-infected mice, leading to reduced bacterial loads and improved survival (212). Collectively, these results provide strong evidence that modulation of type I IFN signaling determines the disease severity and susceptibility of immunopathologic lesions in TB.

#### Th17 Cytokines

Th17 cytokines secreted by Th17 and Th22 cells may play a regulatory role in the immune response during Mtb infection (249). Th17 cytokines play a protective role in Mtb infection but are also involved in pathology due to excessive immune response (250). IL-22 promotes the production of inflammatory mediators and the recruitment of pathologic effector cells in TB (250). IL-22 also promotes tissue repair and healing in lung epithelial cells (251). Treerat et al. demonstrated a significantly increased bacterial burden in the lungs and spleen of *Il22^-/-^* mice (222). Similarly, exogenous administration of IL-22 significantly inhibited intracellular Mtb growth by inducing calgranulin A expression (223). Modulation of Th17 responses is essential to promote anti-TB immunity and block immense immunopathology, leading to a detrimental effect on the host (**Table 2**). Gopal et al. showed that *Il17^-/-^* mice demonstrate increased lung bacterial burden compared to that in wild type mice; however, IL-17 overexpression improved the resistance to TB in Mtb-infected *Il17^−/−^* mice (224). IL-23 induces the production of IFN-γ and IL-17 response that promotes anti-TB immunity. Khader et al. observed increased immunopathological severity in the lungs of *Il23^-/-^* mice (225). The exogenous administration of IL-23 induced significant reduction of the pulmonary Mtb loads and the immunopathological severity in a mouse model *via* enhanced local T cell immunity (226). Taken together, appropriate modulation of Th-17 cytokines is critical to control TB with minimal detrimental effects to the host.

## Conclusion and Future Perspective

Understanding bacterial adaptation to host-mediated stress contributing to antibiotic tolerance is a critical factor in improving disease outcomes and shortening treatment duration. In addition to eliminating the pathogen, effective TB therapy should relieve the associated clinical symptoms by controlling immune-mediated inflammatory responses and minimize damage to the host, thereby minimizing the sequelae. Within the host, Mtb undergoes metabolic reprogramming by drugs or host-mediated stress, resulting in a drug-tolerant state across drug classes. Representative examples of metabolic reprogramming include metabolic stagnation, activation of metabolic bypass, accumulation of triacylglycerol, biofilm formation, and stringent response. The host metabolism is modulated by Mtb infection or the anti-TB drugs, leading to the critical point that determines the treatment success. Therefore, if the host metabolism can be regulated to induce a host-favorable state, the treatment period can be drastically reduced, and side effects can be minimized, dramatically improving clinical outcomes.

## Author Contributions

H-EP, WL, M-KS, and SS wrote the manuscript. M-KS and SS conceived the study, supervised the team, and critically revised the manuscript. All authors contributed to the article and approved the submitted version.

## Funding

This work was supported by the National Research Foundation of Korea (NRF) grant (NRF-2019R1A2C2003204 and NRF-2021R1C1C2012177) and the Bio & Medical Technology Development Program of NRF (NRF-2020M3A9H5104234) funded by the Korean Government (MSIT), Republic of Korea. The funders had no role in study design, data collection and analysis, decision to publish, or manuscript preparation.

## Conflict of Interest

The authors declare that the research was conducted in the absence of any commercial or financial relationships that could be construed as a potential conflict of interest.

## Abbreviations

AhR, aryl hydrocarbon receptor; AMPK, AMP-activated kinase; ARE, antioxidant response element; ARNT, AhR nuclear translocator; ESAT-6, early secretory antigenic target-6; HMG-CoA, β-Hydroxy β-methylglutaryl-CoA; NFκB, nuclear factor kappa B; HO-1, hemeoxygenase-1; ICL, isocitrate lyase; ILIs, intracellular lipophilic inclusions; MIC, minimum inhibitory concentration; Mtb, *M. tuberculosis*; mTOR, mechanistic target of rapamycin; Nrf2-Keap1, NF-E2-related factor 2 (Nrf2)-Kelch-like ECH-associated protein 1; Pht, phthiochol; ppGpp, guanosine pentaphosphate; TA, toxin–antitoxin; TAG, triacylglycerol; TCA, tricarboxylic acid; RNS, reactive nitrogen species; ROS, reactive oxygen species.
